# Vitamin C derivative/AA2P promotes erythroid differentiation by upregulating *CA1*

**DOI:** 10.1093/lifemedi/lnad043

**Published:** 2023-11-13

**Authors:** Xiaoyu Tan, Meng Li, Yue Liang, Xiuyan Ruan, Zhaojun Zhang, Xiangdong Fang

**Affiliations:** Key Laboratory of Genome Sciences and Information, Beijing Institute of Genomics, Chinese Academy of Sciences, China National Center for Bioinformation, Beijing 100101, China; University of Chinese Academy of Sciences, Beijing 100049, China; Key Laboratory of Genome Sciences and Information, Beijing Institute of Genomics, Chinese Academy of Sciences, China National Center for Bioinformation, Beijing 100101, China; Beijing Key Laboratory of Genome and Precision Medicine Technologies, Beijing 100101, China; Key Laboratory of Genome Sciences and Information, Beijing Institute of Genomics, Chinese Academy of Sciences, China National Center for Bioinformation, Beijing 100101, China; University of Chinese Academy of Sciences, Beijing 100049, China; Key Laboratory of Genome Sciences and Information, Beijing Institute of Genomics, Chinese Academy of Sciences, China National Center for Bioinformation, Beijing 100101, China; Beijing Key Laboratory of Genome and Precision Medicine Technologies, Beijing 100101, China; Key Laboratory of Genome Sciences and Information, Beijing Institute of Genomics, Chinese Academy of Sciences, China National Center for Bioinformation, Beijing 100101, China; University of Chinese Academy of Sciences, Beijing 100049, China; Beijing Key Laboratory of Genome and Precision Medicine Technologies, Beijing 100101, China; Institute for Stem Cell and Regeneration, Chinese Academy of Sciences, Beijing 100101, China; Key Laboratory of Genome Sciences and Information, Beijing Institute of Genomics, Chinese Academy of Sciences, China National Center for Bioinformation, Beijing 100101, China; University of Chinese Academy of Sciences, Beijing 100049, China; Beijing Key Laboratory of Genome and Precision Medicine Technologies, Beijing 100101, China; Institute for Stem Cell and Regeneration, Chinese Academy of Sciences, Beijing 100101, China

**Keywords:** AA2P, erythroid differentiation *ex vivo*, *CA1*, metabolism

## Abstract

Vitamin C is used to treat anaemia; however, the mechanism through which vitamin C promotes erythroid differentiation is not comprehensively understood. The *in vitro* erythroid differentiation induction system can reveal the differentiation mechanism and provide erythrocytes for clinical transfusion and anaemia treatment. This process can be promoted by adding small-molecule compounds. In this study, we added l-ascorbic acid 2-phosphate sesquimagnesium salt hydrate (AA2P), a derivative of vitamin C, to an erythroid differentiation system induced from umbilical cord blood haematopoietic stem and progenitor cells *in vitro* and detected its effect on erythroid differentiation using single-cell transcription sequencing technology combined with non-targeted metabolism detection. AA2P increased the proportion of late basophilic erythroblasts, upregulating the expression of erythroid-related regulatory molecules GATA1, KLF1, ALAS2, and the globins HBG and HBB. *CA1* is a target gene of AA2P, and *CA1* knockdown affected the expression of globin-related genes. AA2P also increased glycolysis and decreased oxidative phosphorylation to facilitate terminal erythroid differentiation and enhanced the proliferation of early erythroid progenitors by altering the cell cycle. These results provide a reliable basis for using vitamin C to improve the efficiency of erythropoiesis *in vitro* and for the clinical treatment of anaemia.

## Introduction

The transition from haematopoietic stem/progenitor cells to mature erythrocytes is regulated by multiple factors, including cytokines, transcription factors, and epigenetic modifications at the whole genome and RNA levels. Stage-specific defects may disrupt this process, resulting in abnormal erythropoiesis, and in severe cases, blood diseases [1]. An *in vitro* model of erythroid differentiation can reveal the regulatory mechanisms involved in erythroid differentiation and can serve as a supplementary approach for blood transfusion, aiding in the treatment of anaemia.

In existing *in vitro* erythrocyte regeneration systems, increasing the yield of erythrocytes, improving the expression level of haemoglobin, and promoting the efficiency of enucleation are important issues in this field. Compared with gene transduction, small-molecule compounds have advantages such as ease of control, adjustment, optimization, combination, withdrawal, and low cost. These advantages allow them to accurately control gene expression in cells. They have been demonstrated a strong ability to reprogram cells and significantly increase cell yield as an alternative to gene transduction [2]. Similarly, adding small molecules to the culture environment can help promote the expression of erythrocyte-related proteins and accelerate erythroid differentiation. CHIR99021 [3] and resveratrol [4] have been shown to improve the percentage of burst-forming unit-erythroid (BFU-E) from human pluripotent stem cells and upregulate HBG expression in K562 cell lines.

Studies have found that vitamin C has a strong reducing function, upregulates erythropoietin (EPO) receptors [5], promotes the transferrin Fe uptake [6], and assists in the treatment of iron deficiency anaemia. *In vitro* studies have shown that l-ascorbic acid 2-phosphate sesquimagnesium salt hydrate (AA2P), a derivative of vitamin C, improves enucleation efficiency by neutralizing reactive oxygen species (ROS), which could rescue haematopoietic disorders caused by *IDH1* mutation [7]. However, the effects of vitamin C and its derivatives on erythroid differentiation have not been fully explored; in particular, their effects on metabolism remain unclear.

In this study, single-cell transcription sequencing and metabolome analysis were combined to detect the effect of AA2P on erythroid differentiation. Our study shows that AA2P upregulates various molecules and changes the cell cycle and metabolomics to promote the process of erythroid differentiation.

## Results

### AA2P promotes erythroid differentiation of CB derived CD34^+^ hematopoietic stem and progenitor cells to erythrocytes

**Figure 1.**
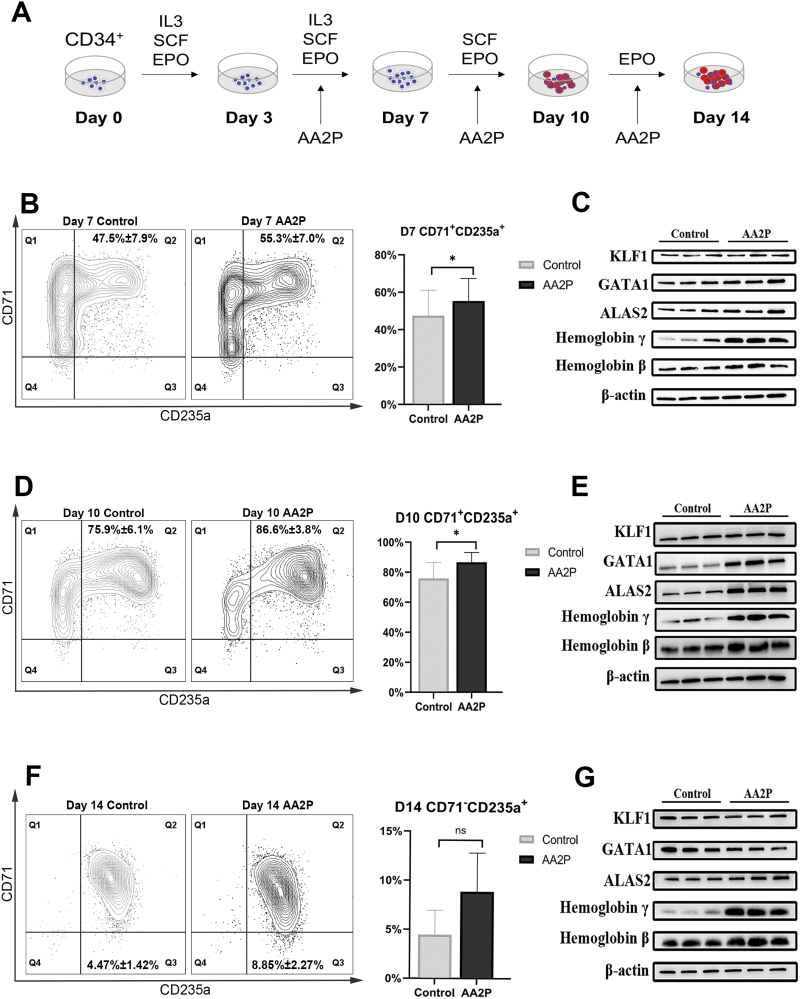
**AA2P promotes erythroid differentiation of CB-derived CD34**^**+**^ **haematopoietic stem cells.** (A) General workflow for *in vitro* differentiation of cord blood-derived CD34^+^ haematopoietic stem cells into erythroid cells. AA2P was added at a concentration of 150 μg/mL on day 3. Gating strategy and quantification of erythroblast populations on day 7 (B), day 10 (D), and day 14 (F) using the surface markers CD71 and CD235a. *n* = 3; bars represent mean ± standard deviation (SD); *P < 0.05, **P < 0.01, and ***P < 0.001 for paired Student’s *t*-test. (C), (E), and (G) Immunoblotting of KLF1, GATA1, ALAS2, hemoglobin β and γ in CB-derived CD34^+^ HSC-induced erythroid cells with or without AA2P at different time points, where β-actin was used as the loading control, *n* = 3.

To detect the effect of vitamin C on erythroid differentiation, we used CD34^+^ haematopoietic stem and progenitor cells derived from human umbilical cord blood (CB) as starting materials. AA2P, a derivative of vitamin C, was added into the culture on day 3 of differentiation induced *in vitro* (Fig. 1A). Based on pilot trials, the appropriate drug concentration was found to be 150 μg/mL (Fig. S1A and S1B). Flow cytometry was used to determine the proportion of CD71^+^CD235a^+^ erythroblasts (EBs). The proportion of CD71^+^CD235a^+^ expressing cells significantly increased from 47.5% ± 7.9% to 55.3% ± 7.0% on day 7 (*P* < 0.05, Fig. 1B), and from 75.9% ± 6.1% to 86.6% ± 3.8% on day 10 in the AA2P treated group (*P* < 0.005, Fig. 1D). The proportion of CD71^−^CD235a^+^ erythroblasts increased from 4.47% ± 1.42% to 8.85% ± 2.27% in the presence of AA2P (Fig. 1F).

GATA1, KLF1, and ALAS2 are important regulatory factors for erythroid differentiation, HBG and HBB are two types of globins. Their expression was detected using quantitate real-time polymerase chain reaction (qRT–PCR) and Western blotting on days 7, 10, and 14 (Fig. 1C, 1E, and 1G). At all three time points, AA2P significantly upregulated the expression of *KLF1*, *ALAS2*, and *HBG* at mRNA level, and *HBG* at protein level compared with those in the control group (Fig. S1C–H). On day 10, the upregulation of *GATA1*, *ALAS2*, and *HBG* of the AA2P group was consistent both at the mRNA and protein levels. These results indicate that AA2P promotes the differentiation of CD34^+^ cells into erythrocytes. The protein level of *HBB* showed an upward trend in the AA2P group on days 10 and 14. The decrease in *HBB* at mRNA level on day 14 may be due to a high proportion of terminally differentiated erythrocytes in the AA2P group with less RNA content and high protein translation efficiency [8].

### Single-cell RNA sequencing (scRNA-seq) data analysis revealed AA2P improves terminal erythroid differentiation

Umbilical cord blood-derived CD34^+^ cells include different types of haematopoietic stem/progenitor cells, that express CD34 and form a mixture of cells containing multiple subtypes at different stages of differentiation when they are induced to differentiate into erythroid cells. scRNA-seq analysis was performed to accurately identify the subsets of cells and genes affected by AA2P; the cells were observed on day 10, when phenotypic changes were most noticeable.

The scRNA-seq library was constructed using a 10 × Genomics platform. Of the 16,379 cells obtained after quality control analysis, 7949 were from the AA2P treatment group and 8430 were from the control group. In total, 6949 and 8933 genes were obtained in the AA2P treatment and control groups, respectively. Clustered cells were based on Seurat's κ-nearest neighbour method, annotated using R package SingleR and the published datasets related to erythroid differentiation. Then they were visualized based on uniform manifold approximation and projection (UMAP). There were 15 cell types in total, and the distribution of cell types in AA2P treatment was consistent with that of the control group (data not shown). The top seven cell types were late basophilic erythroblasts (Late_Baso), pro-erythroblasts (Pro-E), early basophilic erythroblasts (Early_Baso-E), polychromatophilic erythrocytes (Poly-E), colony-forming unit-erythroid (CFU-E), orthochromatic erythroblasts (Ortho-E), and burst-forming unit-erythroid cells (BFU-E).

**Figure 2.**
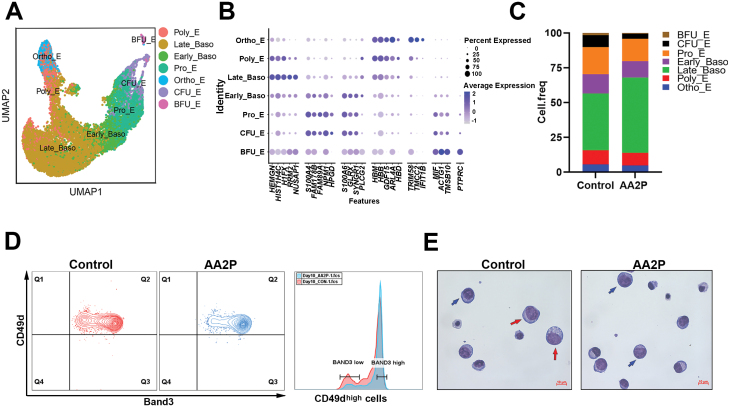
**AA2P improves terminal erythroid differentiation.** (A) UMAP plot of annotation information for single-cell transcriptome data on day 10, categorized by cell type. (B) Top 5 marker gene expression in subgroups. The colour depth indicates the normalized gene expression level, and dot size indicates the proportion of cells expressing the gene in the subgroup. Duplicated genes are deleted. (C) Bar graph showing the proportions of different cell types. (D) FACS analysis of the proportions of cells expressing Band3 and CD49d on day 10. (E) Morphology of erythroid cells on day 10 of *in vitro* differentiation in CB-derived CD34^+^cells. The cells were stained with Wright–Giemsa stain. Red and blue arrows refer to proerythroblasts and late basophil erythrocytes, respectively. Scale bar: 10 μm.

We selected cells from the seven erythroid differentiation types to construct a new dataset and clustered again (Fig. 2A). The FindAllMarkers function was used to analyse marker genes for each cell population. The top five marker genes in each cell population are shown in Fig. 2B [log_2_ fold-change (FC) > 1, value duplicated genes are deleted]. Most cells entered the terminal differentiation stage on day 10 of induction. The scRNA-seq data showed that the largest number of cells in both groups were Late_baso (Fig. 2C), accounting for 40.73% in the control group and 54.36% in the AA2P treatment group. The ratio in the AA2P group increased by approximately 14% compared with that in the control group. However, for the subsets in early erythroid differentiation, including BFU-E and CFU-E, the proportion of BFU-E decreased from 1.4% in the control group to 0.4% in the AA2P group, and the proportion of CFU-E subsets decreased from 8.71% in the control group to 3.6% in the AA2P group. Fluorescence-activated cell sorting (FACS) analysis confirmed that the proportion of cells expressing Band3 at high levels in the AA2P treatment group was higher than that in the control group (Fig. 2D). Giemsa staining revealed more large-sized cells representing at an earlier stage in the control group on day 10 (Fig. 2E), indicating that AA2P improved the terminal differentiation stage.

### Energy metabolism is associated with AA2P facilitating erythropoiesis

**Figure 3.**
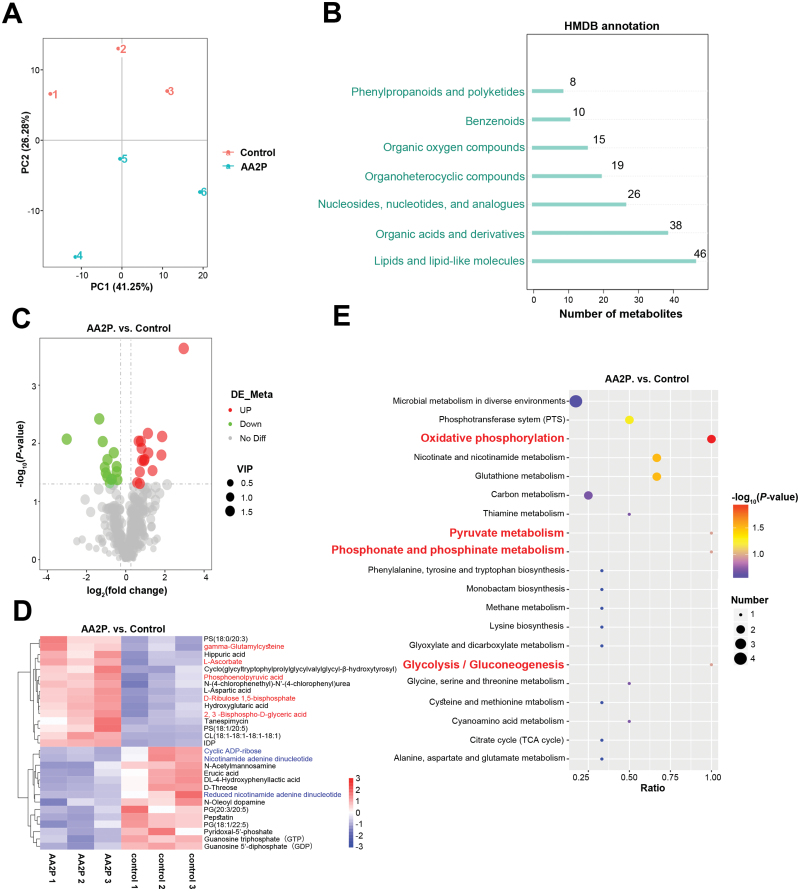
**Energy metabolism is associated with AA2P facilitating erythropoiesis.** (A) PCA plot of the overall distribution trends of the samples. (B) Number of metabolites annotated by Super Class in the HMDB. (C) Distribution trend of metabolites. The abscissa represents the difference in the FC of metabolites in the two groups (log_2_FC); each point in the volcano map represents a metabolite. Red and green dots represent significantly upregulated and downregulated metabolites, respectively, and the size of the dot represents the VIP value. (D) Hierarchical cluster analysis of different metabolites in the two samples: vertical direction is the clustering of samples, and horizontal direction is the clustering of metabolites. (E) Bubble chart of the top 20 differential quantitative metabolic pathways between the control and AA2P treated groups in KEGG pathway analysis. Dot colour represents the *P*-value of the hypergeometric test.

To explore whether AA2P affects metabolic processes during erythroid differentiation, non-targeted metabolomic analysis was performed. We measured six cell samples on day 10: three from the AA2P treated group and three from the control group. After principal component analysis (PCA) for dimensionality reduction clustering, the treated and control groups were well separated (Fig. 3A). Using the Human Metabolome Database (HMDB) for annotation, we obtained seven categories in the secondary classification (Super Class). Of these, the two largest categories were lipids and lipid-like molecules, and organic acids and their derivatives (Fig. 3B). Based on *P* < 0.05 and variable importance in the projection (VIP) > 1.0, 15 metabolites were significantly upregulated and 14 were significantly downregulated (Fig. 3C). Glutathione synthesis intermediates, γ-glutamylcysteine and l-ascorbic acid, were significantly increased, consistent with the metabolites present in cells after adding AA2P; among the increased metabolites in the AA2P-treated group, 2,3-bisphospho-d-glycerate acid (2,3-BPG) and phosphoenolpyruvate (PEP) were both the products of glycolysis. Among them, 2,3-BPG is a specific erythrocyte metabolite that regulates the oxygen affinity of haemoglobin. PEP is converted to pyruvate in the last step of glycolysis and is further converted to acetyl-CoA to enter the citrate cycle [tricarboxylic acid cycle (TCA)], providing succinyl-CoA, a raw material for haeme synthesis and a substrate for the respiratory chain. Compared with those in the control group, nicotinamide adenine dinucleotide (NAD^+^), reduced nicotinamide adenine dinucleotide (NADH), guanosine triphosphate (GTP), and guanosine 5ʹ-diphosphate (GDP) were significantly downregulated in the AA2P-treated group. NAD^+^ connects the TCA cycle and the respiratory chain of oxidative phosphorylation (OXPHOS) and can be degraded to cyclic ADP-ribose (cADPR), another downregulated metabolite in the AA2P-treated group, activating Ca^2+^ signalling. Pyridoxal-5ʹ-phosphate is a coenzyme involved in haeme synthesis (Fig. 3D).

Kyoto Encyclopaedia of Genes and Genomes (KEGG) analysis was performed, and the enriched pathways of differential metabolites included OXPHOS, nicotinate and nicotinamide metabolism, pyruvate metabolism, phosphonate and phosphinate metabolism, glycolysis/gluconeogenesis, and the TCA cycle were identified. Most of these pathways are related to energy metabolism, indicating that AA2P affected energy metabolism (Fig. 3E).

Overall, metabolic analysis revealed that glutathione and energy metabolism, particularly glycolysis, increased in the AA2P-treated group. This may regulate the redox balance and provide energy and raw materials for the synthesis of haeme, which helps cells to enter the terminal erythroid differentiation stage. The relative decrease in the NAD^+^ and NADH indicates that OXPHOS was comparatively weakened in the AA2P-treated group, suggesting that the cells in this group switched from aerobic metabolism to glycolysis more quickly, implying less ROS production.

### CA1 is significantly upregulated by AA2P in erythroid differentiation of CB-derived CD34^+^ cells

**Figure 4.**
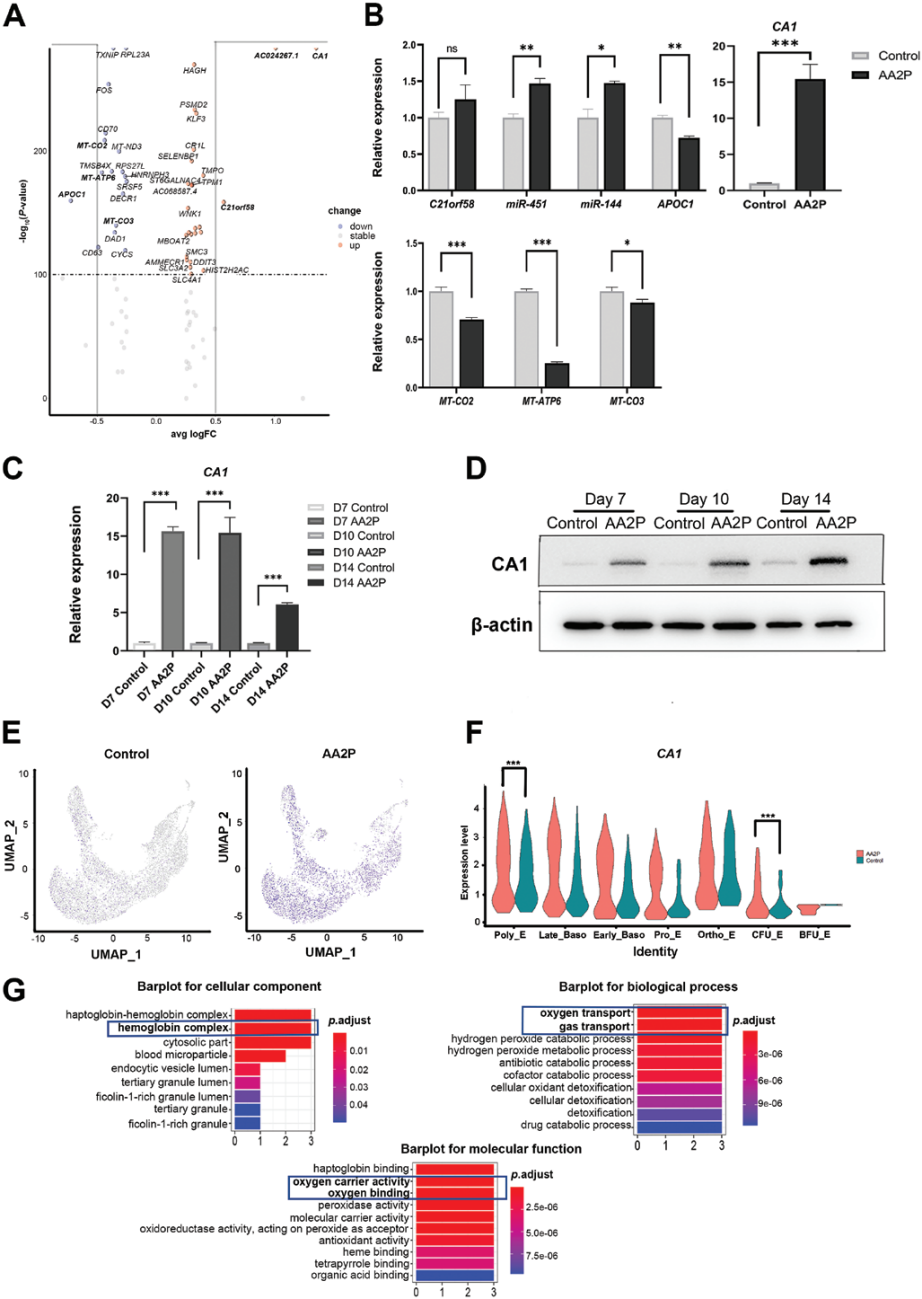
***CA1* is upregulated by AA2P in the process of erythroid differentiation.** (A) Volcano plot depicting DEGs between the AA2P treatment and control groups in the scRNA-seq data on day 10; the abscissa shows logFC, and the ordinate shows the − log_10_(*P*-value). Upregulated genes are underlined in red and downregulated genes are underlined in blue. (B) qPCR results showing the expression of the indicated genes in erythroblasts on day 10. (C) qPCR results and (D) Western blot verifying the expression of CA1 on days 7, 10, and 14. (E) UMAP plot of *CA1* expression in the AA2P treated and control groups. (F) Expression levels of the *CA1* gene in different subgroups based on scRNA-seq (*P*-value). (G) Gene Ontology (GO) annotation based on differentially expressed genes in late basophil erythrocytes enriched in cellular component, biological process, and molecular function.

To identify the genes and signalling pathways influenced by AA2P to promote erythroid differentiation, we analysed the differentially expressed genes (DEGs) using scRNA-seq data of day 10. Cells were divided into control and AA2P groups. There were three upregulated genes (transcripts)—*CA1*, *AC024267.1*, and *C21orf58*, and one downregulated gene—*APOC1*, on the condition of avg_logFC > 0.5 or avg_logFC < −0.5 (Fig. 4A). The qPCR results were consistent with the sequencing data. We observed that *MT-CO2*, *MT-ATP6*, and *MT-CO3* involved in mitochondrial respiratory chain complex, were also downregulated in the AA2P group, suggesting that the OXPHOS of the AA2P group was decreased, which is consistent with our metabolome detection results. Among these, the FC in carbonic anhydrase 1 (*CA1*) was more than 15 times, which has been rarely reported to relate to erythroid differentiation (Fig. 4B). qPCR and Western blotting results confirmed the upregulation of *CA1* in the process of erythroid differentiation upon addition of AA2P (Fig. 4C and 4D). It was upregulated in all cell subsets from BFU-E to Ortho-E, and the differences were significant in the CFU-E and Poly-E subsets (Fig. 4E and 4F; *P*.adjust < 0.001; see Table S1). *miR-451* and *miR-144*, which have been reported to promote erythroid differentiation [9, 10], were located in transcript *AC024267.1* at UCSC (genome.ucsc.edu). These genes were upregulated in the AA2P group. Gene Ontology (GO) analysis of the DEGs in the Late_Baso-E subset based on our scRNA-seq data showed that the oxygen transport, gas transport (biological process), haemoglobin complex (cellular component), and oxygen carrier activity (molecular function) all include *CA1* (Fig. 4G), providing evidence that *CA1* is involved in erythroid differentiation.

### CA1 knockdown significantly affects globin-related gene expression

**Figure 5.**
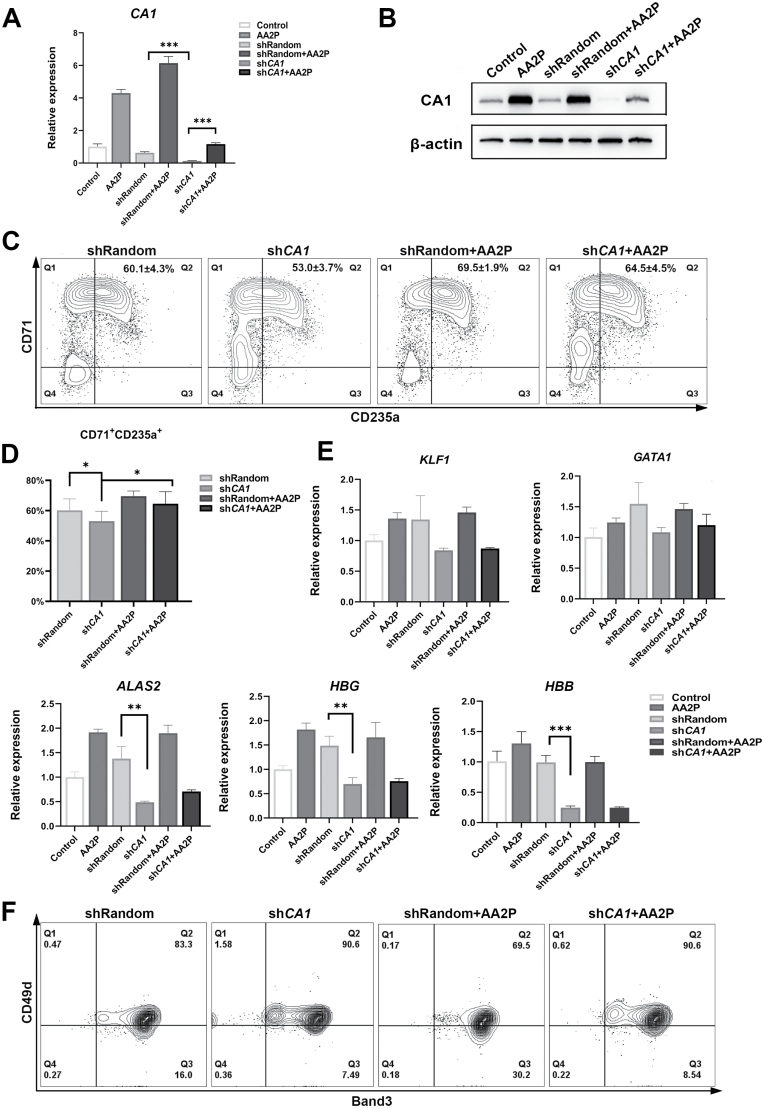
**Knockdown of *CA1* affects erythroid differentiation.** Sh*CA1*-mediated downregulation of *CA1* in CD34^+^ cells was evaluated by (A) qRT–PCR [mean ± standard error of the mean (SEM), *n* = 3] on day 7 and (B) immunoblot on day 10 with β-actin as the native control. (C) FACS analysis and (D) quantification of erythroblast proportions on day 7. (E) qRT–PCR of indicated genes in CD34^+^ cells on day 10 with *ACTB* as the native control. *n* = 3; bars represent mean ± SD; *P < 0.05, **P < 0.01, ***P < 0.001 for paired Student’s *t*-test. (F) FACS analysis of the proportions of cells expressing Band3 and CD49d on day 10. The direction of TED is from CD49d^+^Band3^-^, to CD49d^+^Band3^+^, and then to CD49d^-^Band3^+^(Q1→Q2→Q3).

To identify the role of *CA1* in erythroid differentiation, shRNA-targeted *CA1* was designed and inserted into a lentiviral vector. It was packaged into recombinant lentivirus by human embryonic kidney 293T (HEK293T) cells and then infected with CD34^+^ cells on the second day of differentiation, and AA2P was added to the medium the next day (Fig. S2A). More than 80% of the *CA1* was decreased as analysed by qRT–PCR on day 7 and Western blot on day 10 (Fig. 5A and 5B). The addition of AA2P partly rescued this expression. Compared with that in the shRandom group, the proportion of the CD235a^+^CD71^+^ subset in the sh*CA1* group decreased from 60.1% to 53%, and AA2P addition rescued to the normal levels in the sh*CA1* group on the 7th day of differentiation (Fig. 5C and 5D). The expressions of all the genes of *KLF1*, *GATA1*, *ALAS2*, *HBG*, and *HBB* were downregulated by *CA1* knockdown on day 7 (Fig. S2B). *ALAS2*, *HBG*, and *HBB* were significantly downregulated in the sh*CA1* group on day 10 and could not be rescued by the addition of AA2P (Fig. 5E), indicating that *CA1* upregulation by AA2P may increase the expression of globin-related genes in erythroid differentiation. Globin-related genes decreased by *CA1* knock down could not be rescued by AA2P fully, and we speculated that the erythroid differentiation of cells in the sh*CA1* + AA2P group was still blocked. CD71^+^CD235a^+^ cells contain a variety of cells during the terminal erythroid differentiation (TED) stage. To further distinguish whether there are differences in cells during TED affected by *CA1*, we examined the proportion of cells expressing CD49d and Band3 on day 10 (Fig. 5F). Cells in the Q3 region represent more rapidly differentiated cells such as Poly_E. The ratio of cells in the Q3 region of the shRandom group was 16.0%, and that in the AA2P treatment group increased to 30.2%. *CA1* knockdown reduced the Q3 region ratio to 7.49%, then adding AA2P slightly increased it to 8.54%. This result suggests that *CA1* knockdown affects TED and cannot be rescued by AA2P. This is consistent with the expression trend of globin-related genes in the qPCR results.

### AA2P enhances proliferation of early erythroid progenitor cells by influencing the cell cycle

**Figure 6.**
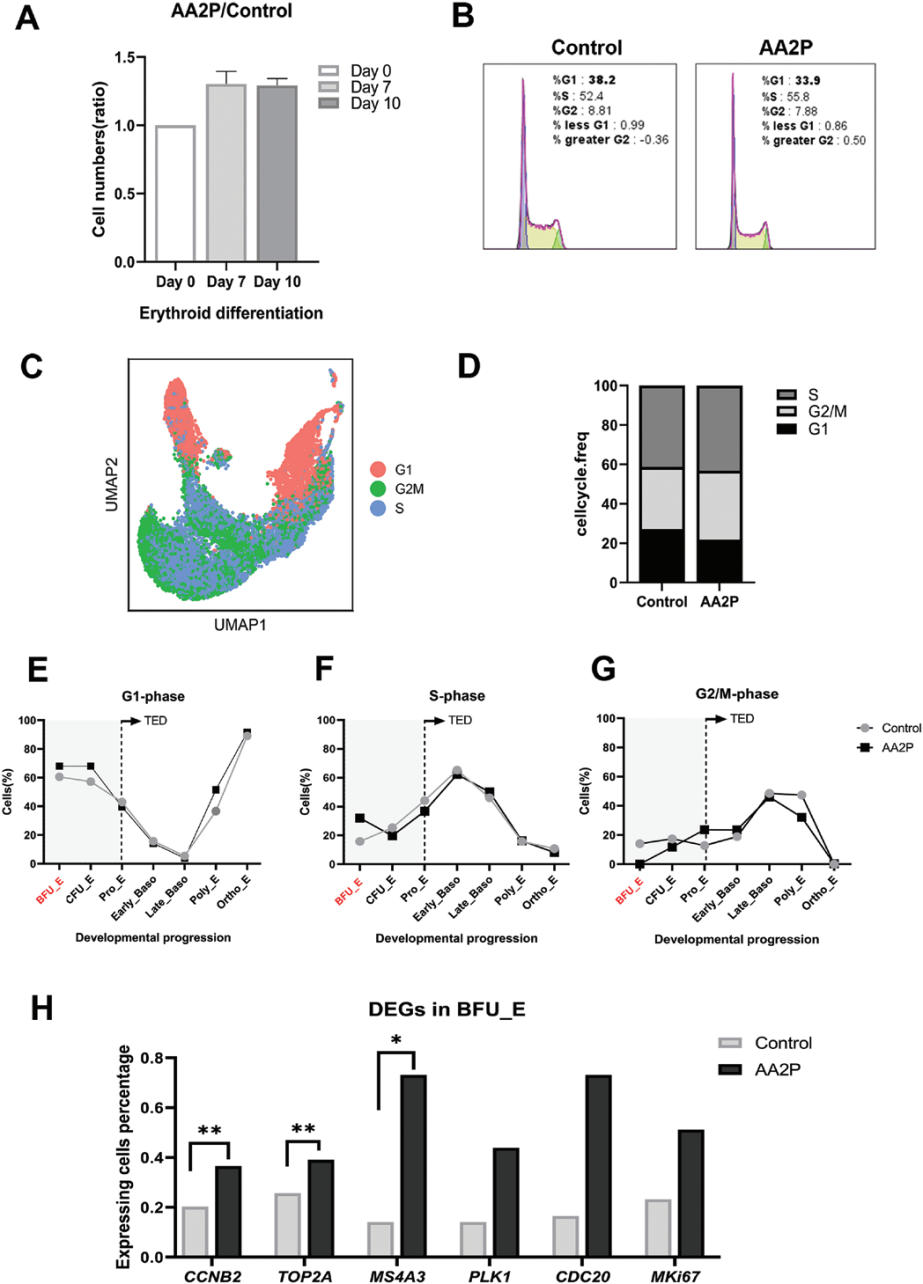
**AA2P enhances cell proliferation by affecting the cell cycle.** (A) Histograms of numbers of cells on days 0, 7, and 10 of differentiation (ratio of AA2P treatment group to control group); *n* = 3. (B) FACS analysis of the cell cycle distribution in CB-derived CD34^+^ cells on day 10 treated with or without AA2P. (C) UMAP plot of annotation information for single-cell transcriptome data on day 10, categorized by classification of cell cycle states of cells associated with erythroid differentiation. (D) Bar graph showing proportions of different cell cycle types. Line charts showing the fraction of cells in G1 phase (E), S phase (F), and G2/M phase (G) along the erythroid trajectory based on the scRNA-seq data, and from Pro-E, cells enter the stage of TED. (H) Percentage of cells expressing proliferation or cell cycle regulating genes in the different expression gene sets of the BFU-E subgroup.

During the induction of erythroid differentiation, we found that AA2P addition increased the cell number by 1.2–1.3 times on both day 7 and day 10 (Fig. 6A). To determine the mechanism through which AA2P promotes cell proliferation, we analysed cell cycle progression. Flow cytometry showed that AA2P treatment decreased the percentage of cells in the G1 phase on day 10 (Fig. 6B) of differentiation. The scRNA-seq data on day 10 were also used to analyse the cell cycle based on the expression pattern of genes, and the results were visualized on a UMAP dimension-reduced map (Fig. 6C and 6D), which suggested that AA2P may promote the G1–S phase transition. In addition, subset analysis showed that in BFU-E, in the early stage of erythroid differentiation, the proportion of cells in the G1 and S phases in the AA2P treatment group was higher than that in the control group (Fig. 6E and 6F), and the proportion of cells in the G2/M phase was significantly lower than that in the control group (Fig. 6G). The expressions of *TOP2A* and *CCNB2* related to the G2/M phase were significantly increased, and those of *CDC20* and *MKi67* also tended to increase; the G1–S phase inhibitor *MS4A3* was significantly upregulated (Fig. 6H). In the early stage of erythroid differentiation, AA2P may accelerate the transition of the G2/M phase.

## Discussion

**Figure 7.**
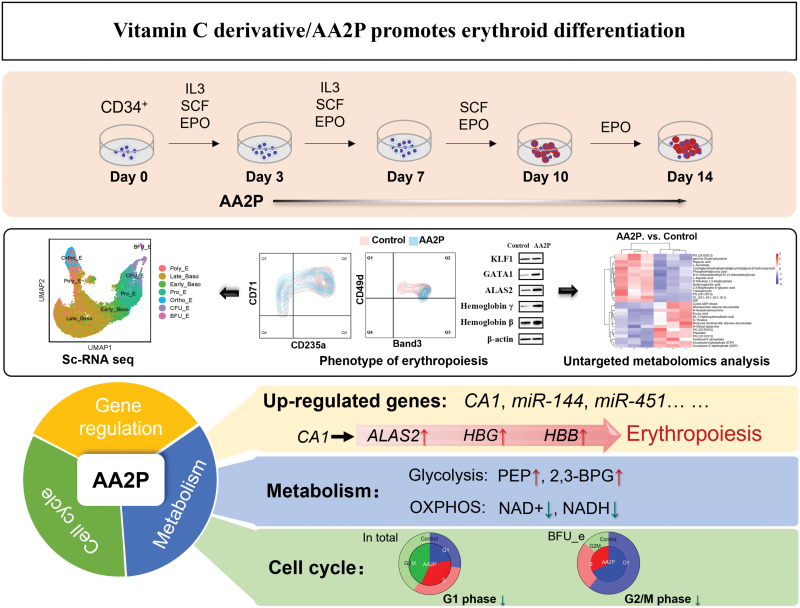
Graphical summary.

This study has four main findings: (i) AA2P can promote the progress of erythroid differentiation and increase the expression of erythroid differentiation-related transcription factors GATA1, ALAS2, and β- and γ-globin chains in haemoglobin at both mRNA and protein levels. (ii) AA2P enhances glycolysis and glutathione metabolism and decreases oxidative phosphorylation in differentiated erythroid cells. (iii) *CA1* is a target gene of AA2P, and knockdown of *CA1* inhibits erythroid differentiation. (iv) AA2P enhances cell proliferation during the early stages of erythroid differentiation (Fig. 7).

*CA1*, a member of the carbonic anhydrase family, is a highly expressed component, second only to haemoglobin, in red blood cells. It forms a complex with haemoglobin to carry and exchange oxygen and carbon dioxide. In the red blood cells of fish and humans, inhibition of *CA1* weakens oxygen-carrying capacity [11–14]. Among the interacting molecules of CA1, there are many key erythroid differentiation-related key proteins, such as GYPB, EPB42, SLC4A1, and ALAS2 present in the STRING database (Version 11). *CA1* can be used in combination with *SLC4A1* and *ALAS2* as an indicator of blood doping [15]. Our data showed a significantly high expression of CA1 in the CFU-E subset of the AA2P-treated group, in which CD71 began to express and haeme was produced in large quantities [16]. During haeme synthesis, many carbon dioxide molecules are released and can be hydrated and transported by CA1. Jing et al. reported that the expression of *CA1* was increasing during erythroid differentiation and reached a peak in the Late_Baso-E subgroup [17], which suggests that the upregulation of *CA1* may contribute to the increase in the Late_Baso-E subset by AA2P, as shown in our results. Our finding of *CA1* knockdown function, which reduced the proportion of the CD71^+^CD235a^+^ subgroup and affected erythropoiesis, is consistent with that of Jing et al. In addition, our results showed that *CA1* knockdown decreased the expression of *ALAS2*, *HBG*, and *HBB*, indicating that *CA1* plays an important role in haeme synthesis and haemoglobin expression. Interestingly, when the knocked down *CA1* was partially rescued by AA2P, the expression of downstream globin-related genes was not still up-regulated, the reason maybe that TED is still blocked.

We found that AA2P upregulated *CA1*, promoting the expression of γ-globin chains in haemoglobin. β-haemoglobinopathies are abnormal haemoglobin HbA caused by the mutation of β6^Glu→Val^. The reactivation of the expression of the γ-globin chain expression has become a viable therapeutic option. From the perspective of gene editing, *BCL11A*, a specific inhibitor of foetal haemoglobin expression [18], has gained significant attention. Editing the enhancer or specific binding loci of *BCL11A* significantly increases *HBG* expression [19, 20]. From a pharmacological perspective, adding compounds such as resveratrol and lipamycin can significantly promote the expression of the γ-globin chain protein [21], and the expression of mRNA and protein in erythroid cells significantly increased after AA2P treatment. Therefore, AA2P may be beneficial in the treatment of β-haemoglobinopathies.

The metabolic remodelling process occurs during erythroid differentiation: in the stem/progenitor cell stage, glycolysis is dominant and OXPHOS is inhibited. When erythroid differentiation is initiated, OXPHOS is activated to satisfy the energy requirement for differentiation [22]. During TED, mitochondrial function is reduced, autophagy occurs, erythroid cells mature [23], and glycolysis is the main metabolic mode of red blood cells. ROS generated from OXPHOS affect TED, particularly enucleation. AA2P promotes enucleation by inhibiting pro-oxidant mitochondrial metabolism [7]. Our study did not focus on the effect of AA2P on enucleation. However, metabolite analysis showed that AA2P enhanced glycolysis and weakened the respiratory chain, thereby decreasing ROS production, which is helpful for TED. The upregulation of γ-glutamylcysteine indicated that glutathione (GSH) synthesis was activated. GSH, particularly in mitochondria, is important for maintaining redox balance during TED [24]. Vitamin C and its derivatives, as key antioxidants, restore the balance in cells with GSH. The molecular mechanisms underlying TED warrant further study. We also observed that the composition of cells during TED differed between the two groups. The proportion of cells in the relative terminal period in the AA2P treatment group was larger than that in the control group. Therefore, changes in metabolism not only reflect the influence of AA2P but also the signature of cells during TED.

Vitamin C can block the cell cycle of tumour cells in the G0–G1 phases but activates cell cycle-related genes in mesenchymal stem cells (MSCs) [25, 26]. However, our results showed that the proportion of cells in the G1 phase of the vitamin C derivative AA2P-treated group was generally smaller than that of the control group on day 10 of erythroid differentiation, indicating that AA2P could promote the G1–S phase transition and accelerate cell proliferation. After analysing the cell cycle of each cell subset at the same time point, more cells were found in the S phase and fewer cells were found in the G2/M phase in the BFU-E of the AA2P treated group. The DEGs data showed that *CCNB2*, which promotes the G2/M phase transition, and *TOP2A*, which regulates chromatin separation and DNA transcription, were significantly upregulated. In addition, *CDC20*, the positive regulator of the spindle assembly checkpoint and the anaphase-promoting complex, and *MKi67*, which represents cell proliferation, showed an upregulation trend, which is consistent with the G2/M phase characteristic molecules in the early stage of erythroid differentiation [27]. These results suggest that AA2P promotes G2/M transition in BFU-E cells. Another significantly upregulated MS4A3 inhibits the G1–S phase transition by binding to CDK2 [28]. It has also been reported via IL-3 signalling that *MS4A3* enhances the endocytosis of beta-strep factor receptors in myeloid cells to promote cell renewal and differentiation [29], demonstrating the diverse functions of *MS4A3*. Promoting cell proliferation in the early stages of erythroid differentiation may be one of the ways through which AA2P enhances erythroid differentiation.

### Research limitations

The process of erythroid differentiation is complex and is affected by cytokines, transcription factors, and inflammatory factors, which are also epigenetically regulated. The current understanding of the mechanisms underlying erythroid differentiation is limited. Although this study clarified the role and molecular mechanism of AA2P in promoting erythroid differentiation, it has some limitations. For example, this study did not adequately analyse how AA2P affects the transcription and translation of the *CA1* gene during erythroid differentiation. Second, this study only examined a single gene of *CA1*, and the regulatory mechanism of AA2P in the erythroid differentiation system is not clear. Finally, target gene mining was not sufficiently extensive.

## Methods

### Research ethics

This study was approved by the Ethics Committee of Beijing Institute of Genomics Chinese Academy of Sciences/China Center for Bioinformation and was performed according to the guidelines of the Declaration of Helsinki. Written informed consent was obtained from each person prior to sample collection.

### In vitro erythroid differentiation induction system

CD34^+^ haematopoietic stem and progenitor cells isolated from human cord blood were purified using a positive-selection magnetic bead system. The cells were amplified in SFEMII medium (STEMCELL) containing 50 ng/mL TPO, 50 ng/mL SCF, and 50 ng/mL FLT3 ligand for 7 days. CD34^+^ cell purity was > 95% after expansion, as determined by FACS. The cells were then induced to differentiate into erythroblasts for 14 days using different cell factors. EPO was added for 14 days at a concentration of 3 U/mL, IL-3 was added for the first 7 days at a concentration of 1 ng/mL, and SCF was added for the first 10 days at a concentration of 10 ng/mL. AA2P (Sigma) was added on day 3 of erythroid differentiation. All cytokines used are listed in the Supplementary Materials.

### Flow cytometry analysis

The cells were analysed for surface expression of CD71, CD235a, and CD49d. We washed 1 × 10^5^ cells twice with Dulbecco’s phosphate-buffered saline (DPBS, Gibco) and suspended them in 50 μL DPBS supplemented with 2% foetal bovine serum (FBS). The cells were then stained with antibodies on ice for 10 min in the dark. They were then washed twice with DPBS and resuspended in 200 μL DPBS for FACS analysis. For intercellular antigen qualification, before staining with the antibody, cells were fixed with 0.05% glutaraldehyde and then permeabilized with 0.01% TritonX-100. All antibodies used are listed in the Supplementary Materials. For cell cycle detection, cells were fixed with 70% ethanol and then incubated with Tali® Cell Cycle solution (Life Technology Corporation). FlowJo (v10.8.1) was used for data analysis.

### Virus production and transduction

The sh*CA1* and random control sequences are listed in Table S2. These were synthesized (Sangon) and inserted into the pSHI-H1-copGFP-T2A-puro vector. The above vectors and three packaged helping vectors (MDLg, rsv-rcv, and vsv-G) were transfected into HEK293T cells. After 72 h, the recommended lentiviruses were harvested from the medium and centrifuged in PEG8000 solution at 3000 rcf for 20 min, and the virus precipitate was resuspended in SFEMII medium. The recommended lentivirus was added to CD34^+^ cells on the second day of differentiation, and the transfection efficiency was determined using FACS to detect the proportion of GFP-expressing cells.

### qRT–PCR

Cells (1 × 10^6^) were lysed in 0.5 ml TRizol (Invitrogen), and the total RNA was isolated according to the manufacturer’s instructions. cDNA was reverse-transcribed using the PrimeScript RT Reagent kit with gDNA Eraser (Takara), and miRNA was reverse-transcribed using a universal primer. qRT–PCR was performed using a Bio-Rad CFX96 real-time PCR detection system (Bio-Rad) with a SYBR FAST Universal qPCR kit (KAPA). Gene expression was normalized to *ACTB* or *U6* levels and reported as FC compared with the control. All primers used are listed in Table S2.

### Western blot

Cells were washed twice with DPBS and lysed in 4% sodium dodecyl sulphate (SDS) at 95°C for 30 min. The cell lysates were centrifuged at 12,000 rcf for 5 min, and the cell concentration was determined using the bicinchoninic acid assay. The proteins were separated by SDS-polyacrylamide gel electrophoresis (Epizyme) and transferred onto a polyvinylidene fluoride membrane (Millipore). Targeted proteins were immunoblotted with the indicated antibodies. β-Actin was used as the native control. All antibodies used are listed in the Supplementary Materials.

### scRNA-seq data analysis

On day 10, 5 × 10^5^ erythroblasts from each group were subjected to chromium 10 × genomic library construction (Annoroad, V3). Raw gene expression matrices generated using CellRanger (v.4.0.0) with default parameter were imported into Seurat (v.3.2.1). Low-quality cells and genes were removed by the condition setting of ‘nFeature_RNA>150&percent.mt<20’, and double/many cell samples were removed using DoubletFinder (v.2.0.3). SingleR (v.1.0.6) and erythroid differentiation sequencing data from Dr. An’s lab [30] were used to annotate the remaining cells. DEGs were identified using the FindAllMarkers command, and functionally annotated DEGs were identified using clusterProfiler (v3.14.3) and org.Hs.eg.db (v3.10.0).

### Untargeted metabolomics analysis

On day 10, three samples of 1 × 10^7^ EBs from each group were harvested and washed twice with DPBS. The metabolites were detected and analysed by Novogene Biotech Co., Ltd. (Beijing, China).

### Wright–Giemsa staining

The 1 × 10^6^ cells in 10 μL FBS were prepared on coated slides. The slides were stained with Wright–Giemsa (Baso) solution according to the introduction. The cells were imaged using a Zeiss Observer Z1 inverted microscope.

### Statistical analysis

All quantitative data are presented as the mean ± SD/SEM. Two-tailed unpaired Student’s *t*-test and one-way analysis of variance were performed using the Prism software (GraphPad Prism V.8.0). *P* < 0.05 was considered statistically significant, and significance is indicated as *P < 0.05, **P < 0.01, or ***P < 0.001.

## Data availability

The raw data of scRNA-seq are available in the Genome Sequence Archive [31] in the National Genomics Data Center, Beijing Institute of Genomics, Chinese Academy of Sciences/China National Center for Bioinformation (GSA: HRA004789) and are publicly accessible at ngdc.cncb.ac.cn/gsa-human/.

## Supplementary Material

lnad043_suppl_Supplementary_Figures_S1-S2

lnad043_suppl_Supplementary_Tables_S1

lnad043_suppl_Supplementary_Tables_S2
