# Supplementary material for: Vitamin C derivative/AA2P promotes erythroid differentiation by upregulating CA1
Source: Life Med. 2023 Nov 13;2(5):lnad043. doi: 10.1093/lifemedi/lnad043 (PMC11749482; doi:10.1093/lifemedi/lnad043)
Supplement: lnad043_suppl_Supplementary_Figures_S1-S2 [file lnad043_suppl_Supplementary_Figures_S1-S2.docx]

**Supplementary Materials**

1. ***Cytokines***

The following cytokines were used for cell and erythroid differentiation: human Flt-3 ligand (Peprotech, 300-19), human TPO (Peprotech, 300-18), human SCF (Peprotech, 300-07), human IL-3 (Peprotech, 200-03-10), and human EPO (PeproTech, 100-64).

1. ***Antibodies***

The following antibodies were used for flow cytometry: PE-CD71 (Miltenyi Biotec), APC-CD235a (Miltenyi Biotec), PE-Band3 (Miltenyi Biotec), and APC-CD49d (BD Biosciences).

The following antibodies were used for western blotting: rabbit anti-human GATA1 (Abcam, ab181544), mouse anti-human KLF1 (Abcam, ab175372), rabbit anti-human ALAS2 (Abcam, ab184964), rabbit anti-human HBG (Abcam, ab137096), rabbit anti-human HBB (Abcam, ab214049), and mouse anti-β-actin (Transgen, HC201-02).


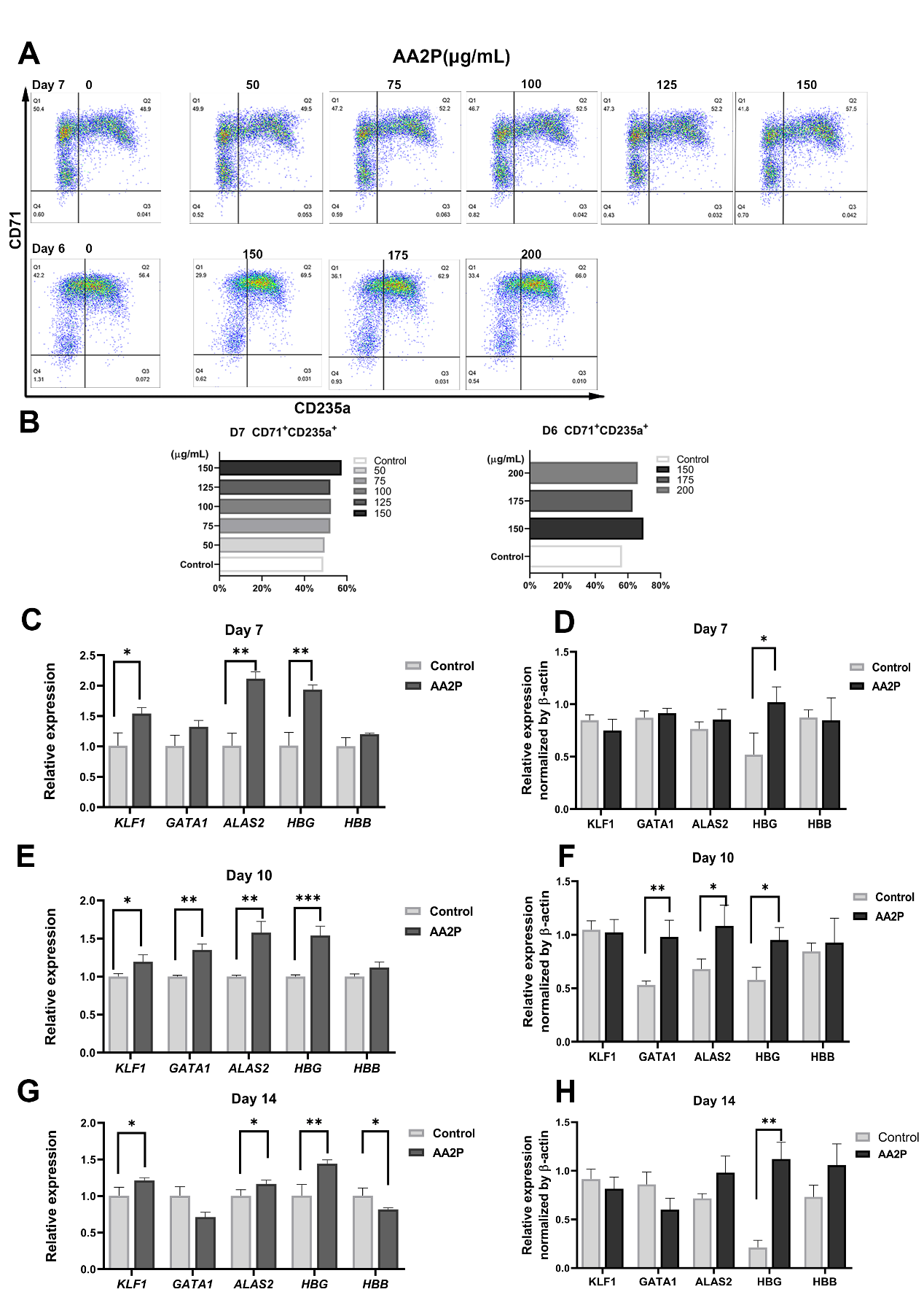


**Figure S1. Pilot experiments to determine a suitable concentration of AA2P.**

1. Fluorescence activated cell sorting (FACS) detection of the proportion of cells expressing CD71 and CD235a of erythroid differentiation with the concentration of L-ascorbic acid 2-phosphate sesquimagnesium salt hydrate (AA2P) from 50 to 150 μg/mL on day 7 (first line) and from 150 to 200 μg/mL on day 6 (second line). (B) Bar chart showing the proportions of CD71^+^ CD235a^+^ cells. Paired t-test significance analysis; **P* < 0.05, ***P* < 0.01; *n* = 3. Quantitative polymerase chain reaction (qPCR) detection (C, E, and G) and western blot analysis (D, F, and H) of the expressions of HBG, HBB, GATA1, KLF1, and ALAS2 in the AA2P-treated and control groups on days 7, 10, and 14. *ACTB* was used as the internal reference gene for qPCR, and β-actin was used as the loading control. The significance was tested using a paired Student’s *t*-test; **P* < 0.05, ***P* < 0.01; *n* = 3.


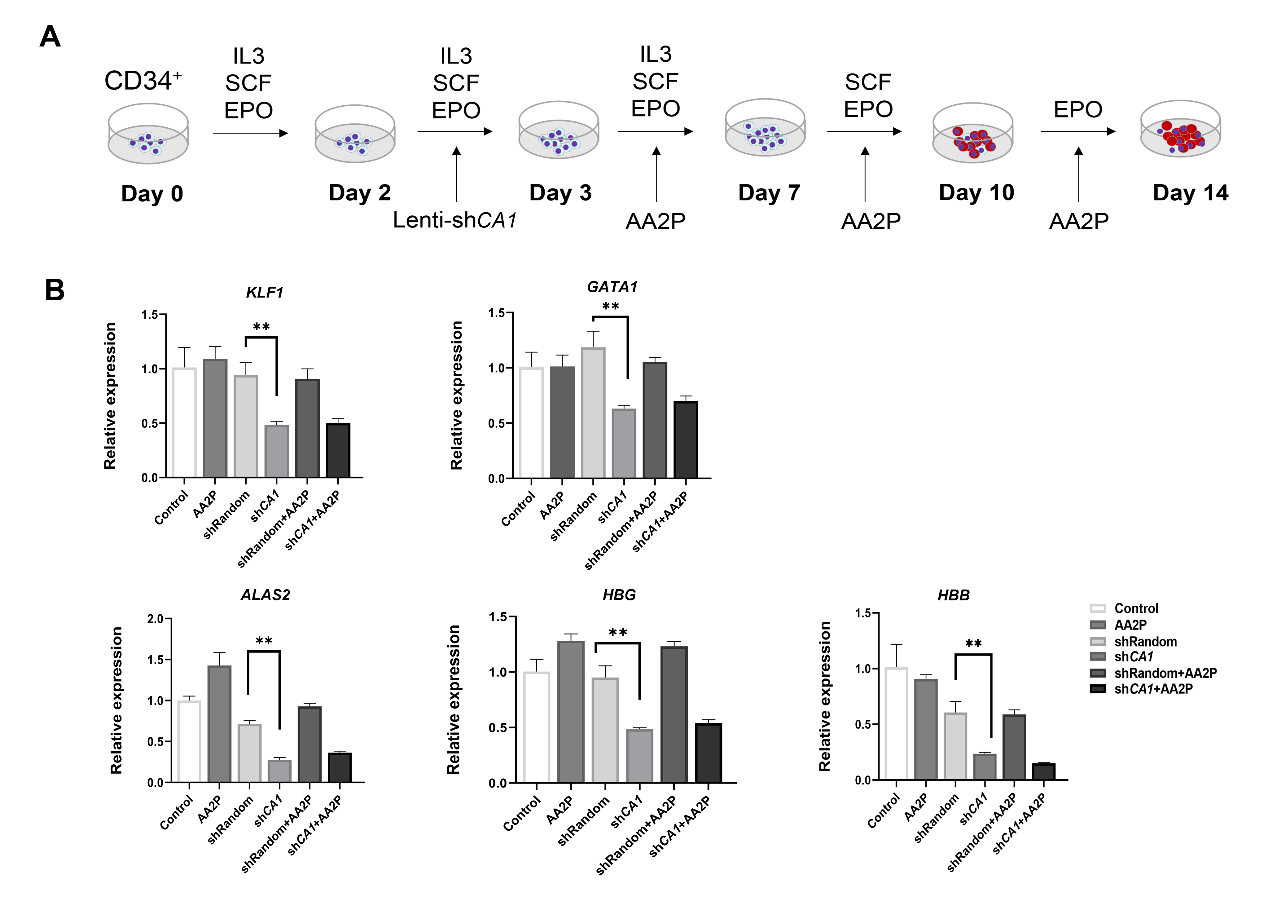


**Figure S2. Knockdown of *CA1* downregulated erythroid differentiation-related genes.**

1. General workflow for the *in vitro* erythroid differentiation system in the condition of carbonic anhydrase 1 (*CA1*) knockdown (similar to Fig. 1A, but for cells infected with recombinant lentivirus containing *CA1* targeted shRNA on day 2). (B) Quantitative real-time polymerase chain reaction (qRT–PCR) of indicated genes in CD34^+^ cells on day 7. *ACTB* was used as the native control. *n* = 3; bars represent mean ± standard deviation (SD); **P* < 0.05, ***P* < 0.01, for paired Student’s *t*-test.

**Supplemental table captions**

**Table S1. DEGs of each subset of cells in scRNA-seq data**

**Table S2. qRT–PCR primer and CA1-shRNA sequences**
